# Battling biofilms: evaluating selected agents against *Cutibacterium acnes*—a review

**DOI:** 10.7717/peerj.20652

**Published:** 2026-01-28

**Authors:** Wala Karar, Seedahmed A. Mohamed, Geetha Subramaniam, Zobidah Yousif Elamin Yousif, Bydaa Atron, Enas dk Dawoud, Harichandra Khalingarajah, Lalita Ambigai Sivasamugham

**Affiliations:** 1 Faculty of Health and Life Sciences, INTI International University, Nilai, Negeri Sembilan, Malaysia; 2 Faculty of Science and Technology, Al-Neelain University, Khartoum, Sudan; 3 Department of Histopathology and Cytology, Omdurman Islamic University, Omdurman, Khartoum, Sudan; 4 Department of Medical Microbiology, Sivas Cumhuriyet University, Sivas, Turkey; 5 Department of Haematology and Immunohematology, Faculty of Medical Laboratory Science, University of Khartoum, Khartoum, Sudan

**Keywords:** *Cutibacterium acnes*, Biofilm formation, Therapeutic agents, Antibiotic resistance, Human health

## Abstract

**Background:**

*Cutibacterium acnes (C. acnes)* is a causative agent in the development of acne vulgaris, and this bacteria has been reported to show resistance against conventional antibiotics. One of the vital factors contributing to antibiotic resistance is the ability of *C. acnes* to form biofilms. Thus, the purpose of this review is to assess the efficacy of various recent developments and to identify acceptable methods for preventing infections associated with *C. acnes* biofilms.

**Methodology:**

A variety of criteria considered in the selection process, such as the site of infection, the mechanism of action against biofilms, and the methodology used to evaluate antibiofilm activity, were taken into consideration when choosing the studies.

**Results:**

The findings of existing research on the antibiofilm potential of conventional anibiotics, natural products and novel treatment strategies against *C. acnes* were compiled and compared. Clinical trials demonstrated that dalbavancin reduced biofilm formation while niosomes effectively decreased inflammation in acne lesions. Some studies have shown promising results with bacteriophages, plant-based and nanomaterial treatments, but lack further validation in the way of pre-clinical and clinical trials to accurately measure treatment effectiveness.

**Conclusions:**

The review examines a range of effective agents and explores their potential applications in acne management, offering valuable insights for clinicians—especially dermatologists—seeking to optimize patient care. In addition, this review provides an understanding about the different agents and their antibiofilm properties that enable researchers to develop effective therapeutic approaches against *C. acnes* biofilm-related infectious diseases for the benefit of human health.

## Background

**Figure 1:**
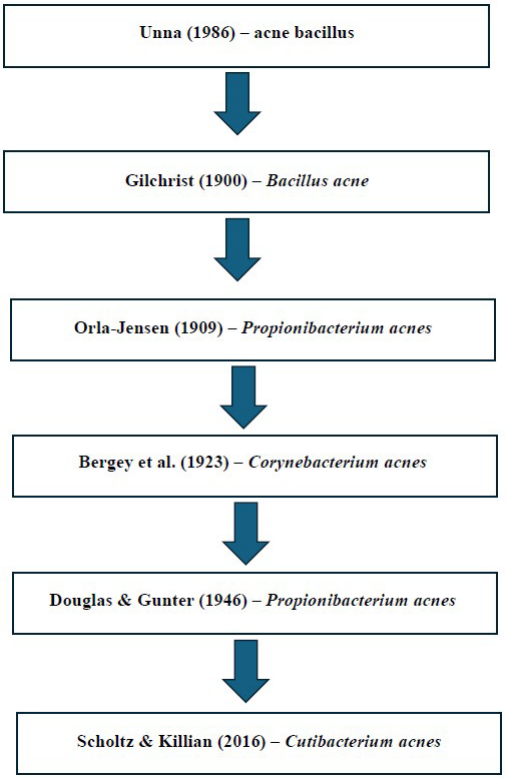
Summary of the taxonomic classification of *Cutibacterium acnes*. Studies: Unna (1986); Gilchrist (1900); Orla-Jensen (1909); Bergey et al. (1923); Douglas & Gunter (1946); Scholz & Kilian (2016).

*Cutibacterium acnes* (*C. acnes*) is a Gram-positive, anaerobic bacterium, and a common member of the skin microbiota, found to colonize sebaceous areas. *C. acnes* was initially observed to be an “acne bacillus” in 1896 by Unna (1986), who discovered this bacterium in the histological sections of acne comedones (Douglas & Gunter, 1946). Later, this bacterium was formally named *Bacillus acne* in 1900 by Gilchrist (1900). However in 1909, Orla-Jensen placed this bacteria under the genus *Propionibacterium* based on its ability to produce propionic acid as one of the end products during the fermentation process (Orla-Jensen, 1909). This bacteria was subsequently placed under the genus *Corynebacterium* due to its morphological relationship to other bacterial species is this group (Bergey et al., 1923). In 1946, Douglas & Gunter (1946) showed evidences of *C. acnes* exhibiting anaerobic metabolism and similar biochemical properties closely resembled those of other species within the genus *Propionibacterium.* One of the shared biochemical properties reported was the ability to ferment lactose into propionic acid. Subsequently, Douglas and Gunter reclassified *Corynebacterium acnes* into the genus Propionibacterium as *Propionibacterium acnes*, based on its characteristic catabolic process in relation to oxygen whereby it produces propionic acid in an oxygen-inhibited environment (Douglas & Gunter, 1946). In 2016, the advent of more comprehensive genome analysis which included 16S rRNA sequences, DNA G+C content, genome size, and peptidoglycan content, revealed significant differences between the cutaneous members of the *Propionibacteriaceae* family which were part of the human skin microbiota, and the classic *Propionibacteria* which were generally isolated from dairy sources. This resulted in the transfer of the cutaneous species into a new genus *Cutibacterium*, resulting in the reclassification of *Propionibacterium acnes* into *Cutibacterium acnes* (Scholz & Kilian, 2016). Based on these findings, the new genus, *Cutibacterium* gen. nov. which accommodated the previous cutaneous species including *Cutibacterium acnes*, *Cutibacterium avidum, Cutibacterium granulosum, Cutibacterium namnetense* and *Cutibacterium humerusii,* was defined (Scholz & Kilian, 2016). In 2020, *Cutibacterium modestum*, a minor member of the skin microbiome, was also included in the genus *Cutibacterium* (Dekio et al., 2021). For easy understanding, the classification of *C. acnes* over time until the present has been summarized in Fig. 1.

Based on the diverse cell wall compositions, *Cutibacterium acnes* can be classified into several ribotypes (Mayslich, Grange & Dupin, 2021), whereby specific ribotypes have been shown to have strong correlation with acne lesions, underscoring the pathogenic potential of select ribotypes (McDowell et al., 2013).

**Figure 2:**
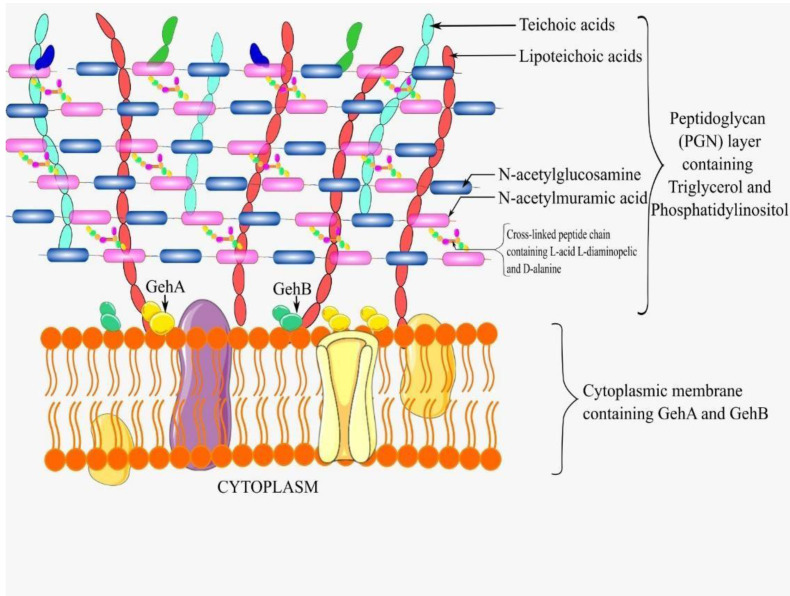
A schematic diagram of *C. acnes* cell wall. Created using Inkscape 1.2 (Inkscape Project. (2020) based on Mayslich, Grange & Dupin (2021), Sagar et al. (2019) and Kaiser et al. (2023).

*C. acnes*, a Gram positive bacteria, has a characteristically thick peptidoglycan (PGN) cell wall layer. The PGN is composed of N-acetylglucosamine and N-acetylmuramic acid, forming a mesh-like structure through cross-linked peptide chains (Mayslich, Grange & Dupin, 2021). Embedded within this layer are teichoic acids, crucial in maintaining ion homeostasis to keep the cell wall integrity intact. The PGN layer in *C. acnes* also contains unique components which include L-acid L-diaminopelic and D-alanine in the peptide layer which are not found in other Gram positive bacteria (Mayslich, Grange & Dupin, 2021). Furthermore, *C. acnes* posseses two unique lipids, phosphatidylinositol and triglycerol, in addition to the common lipids, making the cell wall and envelope distinctive (Jeon et al., 2018). Further analyses have also revealed the presence of a lipid anchor composed of fatty acids and a polysaccharide moeity containing significant amounts of mannose, glucose, galactose, and an amino sugar, likely to be diaminohexuronic acid (Whale et al., 2004). Figure 2 illustrates a schematic diagram of the *C. acnes* cell wall.

The genome of *C acnes* encodes for at least 12 putative lipases (Mayslich, Grange & Dupin, 2021). Out of these, glycerol-ester hydrolase A (GehA), a triacylglycerol lipase, thought to be present on the cell membrane of *C. acnes,* was the first molecule to be identified as a putative virulence factor for this bacteria (Palmieri et al., 2021). These lipases, particularly GehA, digest the sebum on human skin, to release free fatty acids which induce inflammation through the increase in the production of inflammatory cytokines (Li et al., 2017). Lipases facilitate the adherence of *C. acnes* to one another and to other surfaces, hence enhancing their attachment to in-dwelling medical devices such as surgical implants (Scott & Burkhart, 2023).

Initially, *C. acnes* was divided into two serotypes based on serological agglutination tests and the sugar content in the cell wall. Type 1 contained glucose, mannose and galactose, whereas Type II lacked galactose in the cell wall (Johnson & Cummins, 1972).

However, *C acnes* was further divided into phyloptypes I, II and III based on the different cellular properties, biochemical characteristics, immune-inducing abilities and virulence factors, all of which were closely associated to their roles in diseases (Dreno et al., 2024). Using the whole-genome sequencing data, the type I phylotype was further subdivided into four subphylotypes; IA_1_, IA_2_, IB and IC, totaling up to six phylotypes (Corvec et al., 2019; Dréno et al., 2018; Dreno et al., 2024; McLaughlin et al., 2019).

The different *C. acnes* phylotypes have been linked to specific skin conditions. Phylotype IA_1_ is predominantly found in acne-prone skin, while phylotypes IB, II, and III are more common on healthy skin (Dagnelie et al., 2018; Dessinioti & Katsambas, 2024; Dréno et al., 2018). The reduction in phylotype diversity on acneic skin, with IA_1_ being the dominant strain, alters the skin’s microenvironment and promotes inflammation, leading to comedone formation (Dreno et al., 2024). Phylotype IA_1_ is also known to be the most potent producers of biofilm, thus making this phylotype to be more likely to mature into three-dimensional biofilms, sticking to surfaces (Ruffier d’Epenoux et al., 2024).

Other *C. acnes* phylotypes have been linked to other types of infections: IB and II are associated with orthopedic infections, while IB and IC are frequently found in urinary tract and prostate infections (Boisrenoult, 2018; Mayslich, Grange & Dupin, 2021).

One important aspect of *C. acnes’* pathogenicity is its ability to develop biofilms. Biofilms aid in the development of resistance towards antibiotics, which make infections challenging to treat using these therapeutic agents. In addition, biofilms shield the bacteria from environmental stress and the host immune system, this increasing the survival of these pathogens (Mayslich, Grange & Dupin, 2021). Due to these reasons, it is vital to conduct research on the processes of biofilm formation since antibiotic-resistant *C. acnes* infections are increasingly found among implantable medical devices.

This narrative review provides a comprehensive analysis of the biofilm-forming capabilities of *C. acnes* and the implications for antibiotic resistance. This review also explores the impact of biofilm-related infections on implantable devices and discussing recent advances in therapeutic strategies aimed at enhancing antibiotic efficacy. In addition, this review explored some potential therapeutic avenues that could lead to more effective management of *C. acnes*-associated infections, particularly those complicated by biofilm formation.

## Survey Methodology

This narrative review systematically examined existing research on *C. acnes* biofilm development in medical implants, antibiotic resistance in *C. acnes* and therapeutic interventions to minimize this issue. A comprehensive literature search was conducted using databases like NCBI, Google Scholar, and Research Gate, with inclusion and exclusion criteria outlined in Table 1. The methodology for data collection in this review involved systematically extracting relevant information from each primary study related to the anti-biofilm activity and *C. acnes*, included in the sample, focusing on information pertinent to the topic. Subsequently, the review team collated, summarized, and organized, the extracted evidence to synthesize a comprehensive understanding of the topic.

**Table 1:** The inclusion and exclusion criteria that have been used for this review paper.

Inclusion Criteria	• Papers on *Cutibacterium acnes* published in SCOPUS or WoS journals within the past 10 years • Papers that included information about taxonomy, cell wall composition, antibiotic resistance, biofilm development, anti-biofilm activity, *etc.*
Exclusion Criteria	• Papers including information solely belonging to other genera of Gram negative bacteria. • Papers published in non-SCOPUS journals.

## *C. acnes* Biofilms and Related Infections in Implant-Associated Devices

The ability of *C. acnes* to form biofilms was first described by Tunney et al. (1999), who found this on prosthetic hip implants. Subsequent studies demonstrated that biofilm formation by *C. acnes* occurred on a variety of other implants and highlighted the detrimental effects of this characteristic on treatment options, including antibiotic therapy (Rieger et al., 2013; Rieger et al., 2014).

Biofilms are complex communities of bacteria encased in a self-produced matrix of substances like polysaccharides, proteins, and DNA. This matrix creates a unique microenvironment with gradients of nutrients, oxygen, and pH, that provides a niche which shields bacteria from external threats, including antibiotics (Uruén et al., 2020). This protective layer cause the bacteria to become significantly more resistant to antimicrobial agents compared to their planktonic counterparts. Biofilms provide a safe haven for bacteria to survive and multiply, thus becoming reservoirs for recurrent infections leading to chronic and persistent infections even after treatment (Wood et al., 2012; Sharma et al., 2024).

*C. acnes* biofilm production is characterized by the increased secretion of virulence factors such as lipases compared to the planktonic version of *C. acnes* (Coenye, Peeters & Nelis, 2007). The biofilms pose a significant challenge to antibiotic therapy by hindering drug absorption and action efficacy, which is one of the main drug mechanisms (Dessinioti & Katsambas, 2017; Mayslich, Grange & Dupin, 2021).

The production of biofilms by *C. acnes* is especially alarming in relation to orthopedic implant-associated infections and other device-related problems. Multiple factors can affect the capacity of *C. acnes* to develop biofilms on medical devices including implant surfaces, bone microenvironment and bacterial internalization (Varin-Simon et al., 2025). Different implant materials such as titanium alloys and polymers, have different surface roughness that influences biofilm formation of *C. acnes*. The presence of osseous tissue can influence biofilm properties, with some strains known to modify adhesion and biofilm composition when subjected to bone microenvironments. This was demonstrated in a study by Mongaret et al. (2020) who showed that commensal *C. acnes* strains significantly increased the biofilm formation (2.8-fold) after internalization into bone cells, an effect that was further amplified on titanium surfaces. Different phylotypes of *C. acnes* also demonstrate differing capacities in biofilm formation. Using microtiter plate experiments, Kuehnast et al. (2018) showed that IA_1_ isolates exhibited the largest quantities of biofilm, followed by isolates of the IC, IA_2_, and II phylotypes. Another interesting finding was that a community comprising of two distinct phylotypes of *C. acnes* that displayed unique transcriptional profiles exhibited enhanced biofilm formation compared to a single-strain community (Bjerg et al., 2024). A recent report showed *C. acnes* isolates from a deep seated infection in a prosthetic joint belonged to the IA_1_ phylotype, indicating this particular phylotype’s ability to cause invasive infections (Both et al., 2023).

*C. acnes* have been found to form biofilms on many prosthesis-related materials, including polymethylmethacrylate bone cement as well as the materials used in prothesis fabrication such as titanium alloys (TiA), cobalt-chromium-molybdenum (CoCrMo), polyether ether ketone (PEEK), and stainless steel (Bidossi et al., 2020; Garcia et al., 2020; Ramage et al., 2003). The formation of biofilms on medical devices often leads to chronic infections and implant failures, particularly in older populations where neurosurgical implants are common. This issue is gaining prominence, highlighting the urgent need for effective strategies to counter biofilm-related complications (Caldara et al., 2022; Gharamti & Kanafani, 2017; Conen et al., 2020).

In addition, *C. acnes* has been found to be a major cause of cerebrospinal fluid (CSF) shunt infections. Diagnosing *C. acnes* infections can be challenging due to difficulties in culturing the bacterium (Prozan et al., 2020; Beaver et al., 2021). Elevated inflammatory markers were observed in both CSF and brain tissues of rats inserted *C. acnes*-infected catheters (Beaver et al., 2021). Additionally, infections of postoperative central nervous system (CNS) and cranial neurosurgeries are frequently associated with *C. acnes*, emphasizing its importance as a causative agent (Prozan et al., 2020; Strahm et al., 2018).

*C. acnes* has also been implicated in infections related to cardiovascular implantable electronic devices (CIEDs), representing a significant portion of atypical pathogen cases. Strains isolated from device surfaces have shown a propensity to form biofilms, contributing to persistent infections (Kohli et al., 2021; Okuda et al., 2018). Furthermore, *C. acnes*-infective endocarditis cases often involve prosthetic valve endocarditis or infections associated with annuloplasty rings (Banzon et al., 2017). These findings underscore the necessity to improve diagnostic methods and to increase awareness of *C. acnes* infections, particularly in the context of CNS and cardiovascular device-related infections.

*C. acnes* has also been found to be a causative agent in chronic breast implant infection and related capsular contractures complication. This could lead to post-operative infectious complications which are among the leading causes of surgical readmission (Hanna et al., 2023). In orthopedic settings, the Debridement Antibiotic Pearls and Retention of the Implant (DAPRI) technique aims to combat infections by removing intra-articular biofilm using antibiotic-loaded beads, resulting in a high success rate in preventing periprosthetic joint (PJI) infections (Indelli et al., 2023). An example is using synthetic calcium sulfate beads loaded with antibiotics which efficiently inhibit bacterial growth, preventing and minimizing biofilm formation—the main factor contributing to periprosthetic infections. Although antibiotic-loaded beads have the potential to reduce biofilms, total eradication is still difficult, highlighting the importance of early intervention to stop infection (Howlin et al., 2015). In a related study, using combined titanium implants with local drug delivery systems have shown promise in enhancing orthopedic therapies by improving antibacterial effects and stimulating osseointegration (Ma et al., 2021). These novel methods of treating biofilm-related infections in patients with surgical implants could improve the overall outcome of the patient by reducing such complications.

Nonetheless, future research should prioritize conducting *in vivo* studies to better replicate clinical conditions, as current investigations are predominantly limited to *in vitro* environments that may not fully capture the complexity of biofilm-associated infections. A deeper understanding of the genetic and biochemical pathways involved in biofilm formation and maintenance is essential, which can be achieved through multi-omics approaches—including genomics, transcriptomics, proteomics, and metabolomics—to comprehensively unravel host-pathogen interactions and microbiome dynamics. Additionally, developing sophisticated animal models and *ex vivo* systems that closely mimic human skin and implant environments can provide more physiologically relevant platforms for testing therapeutic interventions.

In addition, despite the major advances in therapeutic options, the treatment of biofilm-related infections is further complicated by the increase in antibiotic-resistance among the biofilm-forming *C. acnes* isolates.

## Antimicrobial Resistance in *C. acnes*

The first antibiotic resistance case in acne was observed in the 1970s and involved *C. acnes* isolates (Pannu et al., 2011). In the 1980s, the cases of antibiotic resistance in acne increased to a point that they became a main concern in dermatology (Humphrey, 2012). Studies have highlighted varying levels of antibiotic resistance among *C. acnes* strains isolated from acne patients from diverse geographic regions, underscoring the global significance of this issue. Given that biofilm formation has been observed in acne samples, understanding the antibiotic resistance profiles of these isolates is crucial. Table 2 summarizes data from studies conducted worldwide on antibiotic resistance among *C. acnes* isolates.

**Table 2:** Incidence of antibiotic resistance among dermatology patients from various countries.

**Country**	**Sample size**	**% of resistance to antibiotics**	**Year of publication**	**Reference**
Thailand	143	Trimethoprim/sulfamethoxazole (100%) Clindamycin (75.5%) Erythromycin (73.4%) Tetracycline (51.7%) Doxycycline (51.1%)	2023	Sermswan et al. (2023)
Colombia	129	Tetracycline (5.43%) Doxycycline (5.43%) Minocycline (0.78%)	2021	Castellanos Lorduy et al. (2021)
Israel	50	Erythromycin (25.0%) Clindamycin (16.7%) Doxycycline (19.4%) Minocycline (11.1%) Tetracycline (8.3%)	2020	Sheffer-Levi et al. (2020)
Jordan	100	Erythromycin (73%) Clindamycin (59%) Doxycyclin (37%) Tetracycline (36%) Trimethoprim / Sulfamethoxazole (31%) Levofloxacin (15%) Minocycline (3%)	2020	Alkhawaja et al. (2020)
Singapore	402	Clindamycin (27.2%) Erythromycin (26.8%) Tetracycline (6.0%) Doxycycline (9.4%) Minocycline (1.7%)	2019	Jha et al. (2019)
China (Shanghai)	63	Clindamycin (28.6%) Erythromycin (49.2%) Tetracycline (0%) Morfloxacin (6.3%) Minocycline (1.7%)	2019	Zhang et al. (2019)
China (Southwest China)	97	Clindamycin (30.1%) Azithromycin (28%) Erythromycin (26.9%) Tetracycline (0%) Doxycycline (0%)	2019	Zhu et al. (2019)
India	80	Clindamycin (90.4%) Azithromycin (28%) Erythromycin (26.9%) Tetracycline (0%) Doxycycline (0%) Minocycline (1.9%)	2016	Sardana et al. (2016)

Based on Table 2, data collected from various regions showed that *C. acnes* consistently exhibited resistance to clindamycin and erythromycin possibly due to the frequent usage of both antibiotics for treatment of acne vulgaris (Coates et al., 2002), who noted that these isolates were resistant to both these antibiotics in the samples tested. The data analyzed showed that in India *C. acnes* had the highest resistance to clindamycin which accounted for 90.4% of the patients’ samples analyzed. The data in Table 2 also showed that *C. acnes* developed the highest resistance against erythromycin particularly among the patients from Jordan and Israel. *C. acnes* isolated from Thailand, India, and Jordan, exhibited equally high resistance to multiple antibiotics, highlighting the urgent need for effective treatment strategies. Particularly alarming is the increasing resistance to newer antibiotics like doxycycline and minocycline, which if not kept in check, will limit the already restricted treatment options.

Based on data from 39 studies conducted globally, there is a marked increase in resistance towards erythromycin and trimethoprim-sulfamethoxazole (Beig et al., 2024). The antibiotic susceptibility profiles of the *C. acnes* differ in resistance patterns due to the varied antibiotic prescribing regime of the individual countries.

Given the limitations of conventional antibiotic therapy in treating *C. acnes*, studies have explored various strategies to enhance antibiotic efficacy, including synergistic combinations and alternative agents. Using a combination of antibiotics with different mechanisms of action could enhance treatment efficacy. For instance, combining antibiotics with agents that disrupt the biofilm matrix could improve drug penetration to erradicate biofilm-forming bacteria (Bari et al., 2023). Developing agents specifically targeting the biofilm matrix or the biofilm-forming process in *C. acnes* can prevent biofilm formation or promote biofilm dispersal. Enzymes that degrade extra polymeric substances (EPS) components within the biofilm or molecules that inhibit quorum sensing are potential candidates in these strategies (Sikdar & Elias, 2020). Using combined antibiotics like rifampin with β-lactams or clindamycin, and combinations of antibiotics with calcium sulfate, or with povidone-iodine have been shown to successfully treat periprosthetic joint infections caused by biofilm-associated *C. acnes* (Kusejko et al., 2021). A clinical trial in USA showed that dalbavancin, a long-acting lipoglycopeptide, was effective in reducing biofilm formation by other similar Gram positive bacteria (Goodman-Meza et al., 2025), highlighting the potential of this agent as a promising antibiotic against *C. acnes* biofilms which merits further investigation (Gatti et al., 2021).

On top of developing new agents to combat antibiotic resistance in *C. acnes*, continuous monitoring of antibiotic resistance patterns particularly to the front-line acne antibiotics such as clindamycin, erythromycin, and doxycycline, should be implemented through global and regional surveillance networks. These surveillance efforts must extend beyond traditional approaches by studying the dynamics of resistance development within biofilms *versus* planktonic cells, as biofilm-associated bacteria often exhibit distinct resistance mechanisms and profiles compared to their planktonic counterparts. Furthermore, investigating the impact of antibiotic stewardship programs on resistance rates in dermatological practice can provide valuable insights into how prudent antibiotic usage can slow or prevent the emergence of resistant strains, ultimately contributing towards evidence-based prescribing guidelines and preserving the efficacy of existing therapeutic options.

## Natural Agents Inhibiting *C. acnes* Biofilm Formation

With the increasing incidence of antimicrobial resistance, researchers are looking towards alternative antibacterial agents to inhibit biofilm formation and subsequently curb bacterial infections. Medicinal plants and other natural compounds have been the focus of such research, offering novel therapeutic options to address antimicrobial resistance (Himanshu et al., 2023; Kong et al., 2022). Some of these studies have been summarized in Table 3.

**Table 3:** Examples of natural compound exhibiting anti-biofilm activity against *C. acnes*.

**Natural compound**	**Example**	**Main phytochemicals involved**	**Mode of action**	**Reference**
**Algae**	Ulva seaweed	Ulvan	Inhibits biofilm formation	Fournière et al. (2021)
	*Arthrospira platensis*	Free-fatty acids (in alginate-based nanocarriers)	Targets matrix of biofilms	Lemoine et al. (2020)
**Plants**	*Helichrysum odoratissimum*	*α*-humulene, *α*-curcumene, caryophyllene	Reduces adherence to surface preventing biofilm formation.	De Canha et al. (2020)
	*Myrtus communis*	myrtucummulones, ursolic acid	Alters chemical compounds involved in biofilm development	Ruffier d’Epenoux et al. (2024)
	*Sapindus mukorossi Gaertn.*	saponins	Decreased adhesion and cell surface hydrophobicity	Wei et al. (2021)
	*Arctostaphylos uva-ursi *	arbutin	Inhibits and degrades biofilm matrix	Dell’Annunziata et al. (2022)
	*Zingiber officinale, Juglans regia*	zingiberene, gingerol	decreased production of extracellular polymeric substances	Silva et al. (2022)
	*Thymus*×* citriodorus* (Pers.) Schreb*.*	geraniol, 1,8-cineole, thymol	Dirupts biofilm formation	Oliveira et al. (2022)
	*Lithospermum erythrorhizon*	shikonin	Reduces attachment to surfaces and downregulated expression levels of quorum-sensing regulator genes	Kim et al. (2024)
**Bacteria**	*Bacillus circulans*	glucose	Glucose–*B. circulans* co-culture enhanced electricity production and significantly supressed *C. acnes* growth	Kao et al. (2022)

Algae, particularly red algae, are rich in bioactive compounds like polysaccharides and terpenoids, exhibiting potent *in-vitro* anti-inflammatory effects. However studies regarding the antibacterial activity of these Rhodophyta sp. against *C. acnes* and *S. epidermidis* are limited (Januário et al., 2021). Green macroalgae have also been shown to exhibit antibacterial activity against *C. acnes*. Polysaccharide and oligosaccharide fractions from *Ulva* sp. at 1,000 µg/mL were shown to reduce the inflammation on both acneic and non-acneic *C. acnes* strains on keratinocytes (*in-vitro*) by up to 39.8%, demonstrating promising biological activity for potential dermo-cosmetic applications (Fournière et al., 2021). However, before these compounds can be considered as potential cosmeceutical products, these compounds need to be assessed for long-term safety, and compatibility with human skin. Further investigations especially on crude plant extracts with regards to biofilm development in *C. acnes*, and subsequently, pre-clinical and clinical trials are required.

Medicinal plants have been used throughout the centuries as therapeutic agents against various ailments particularly infections. These category of plants are rich in phytochemical compounds such as alkaloids, flavonoids, and tannins, which exhibit antimicrobial properties effective against a broad spectrum of pathogens, including those forming biofilms. The following examples highlight medicinal plants that have shown antibacterial effects against *C. acnes.*

*Helichrysum odoratissimum* combats acne by directly inhibitng the growth of *C. acnes* and also the formation of biofilm where the extract exerted a strong anti-adherence effect (De Canha et al., 2020). *Callicarpa americana* leaf extracts inhibit the growth of *C. acnes*, whereby 30–40% drop in biofilm formation was observed (Pineau et al., 2019). *Humulus lupulus*, or Hop extract was shown to inhibit *in vitro* biofilm formation of multidrug-resistant *C. acnes*, thus indicating this plants promising usage to treating skin conditions including acne (Di Lodovico et al., 2020).

Traditional medicinal plants like black ginger, *Cotoneaster* species, and *Psidium guajava* were reported to contain antimicrobial properties against *C. acnes* in *in vitro* experiments. Among the three, *Cotoneaster* species extracts not only exhibited antibacterial activities against *C. acnes*, but also antibiofilm properties and are non-cytotoxic (Krzemińska et al., 2022; Sitthichai et al., 2022).

Propolis, a resinous secretion from bees, is a substance rich in plant-derived phytochemicals. Propolis has been shown to exhibit significant anti-biofilm activity against several skin pathogens including *C. acnes* by downregulating genes essential for bacterial attachment and colonization, while maintaining the natural biodiversity of the skin microbiome (Athanasopoulou et al., 2025).

*Psidium guajava*, although not studied extensively, possesses phytochemicals with potential antibiofilm mechanisms (Gutierrez-Montiel et al., 2023). Witch hazel (*Hamamelis virginiana* l.), while lacking direct antibacterial or antibiofilm effects against *C. acnes*, possesses potent anti-inflammatory properties, that could alleviate acne symptoms (Piazza et al., 2022).

Essential oils (EO) from various plants have extensive industrial applications, with thyme EO showing potent bactericidal and antibiofilm effects against *C. acnes* and *S. epidermidis*, attributed to thymol (Abdelhamed et al., 2022; Zhang et al., 2022). Oregano EO exhibits similar potency, while tea tree EO demonstrates synergistic effects with other constituents (Bungau et al., 2023; Nascimento et al., 2023). Green tea extracts inhibit *C. acnes* biofilm formation by modulating autoinducer 2 (AI-2) production (Cañellas-Santos et al., 2023).

While essential oils have shown promising antimicrobial properties, they also have several disadvantages, whereby the antimicrobial activity of essential oils can vary depending on factors such as plant species, geographic origin, extraction method, and storage conditions. Furthermore, essential oils often exhibit a narrow spectrum antimicrobial activity. Volatility, skin sensitivity, interactions with medications, and resistance development are some of the factors that should be considered when working with essential oils as potential antimicrobial agents (Swamy, Akhtar & Sinniah, 2016).

These *in vitro* studies highlight how plant-derived compounds offer promising alternatives to combat microbial infections and biofilm formation effectively. However, further investigations using *in vivo* models should carried out before actual therapeutic application can be achieved.

Other natural antimicrobial compounds derived from bacterial cells represent another promising alternative. Certain bacteria can produce metabolites and bioactive substances capable of inhibiting *C. acnes* growth and disrupting biofilm formation.

*Bacillus circulans* fermentation with glucose showed promising results in inhibiting *C. acnes* growth and biofilm formation through the generation of Kao et al. (2022). In an *in vivo* study, *C. acnes* culture was injected into the ears of mice to induce an inflammatory response. The subsequent administration of *B. circulans* fermentation culture resulted in an increased electrical production, leading to a reduction in *C. acnes* population, demonstrating a novel way of treating bacterial acne infections (Kao et al., 2022).

Recent research on a marine actinobacteria *Promicromonospora* sp. extract showed strong quorum quenching activities against various bacterial species including skin pathogens such as *S. epidermidis* and *S. aureus* (Hoz-Romo et al., 2025). In addition, the *Promicromonospora* sp. extracts also exhibited anti-biofilm and anti-oxidant activites, which could be beneficial as a potential therapeutic option for skincare management.

Microorganisms are also known to secrete biosurfactants, that possess the ability to reduce interfacial tension and inhibit biofilm formation. A glycolipid biosurfactant from the *Acinetobacter* M6 strain effectively reduced biofilm formation by 82.5% in MRSA bacteria (Karnwal et al., 2023). The possibility of using biosurfactant as an antimicrobial agent to combat *C. acnes* infections should be further researched. Similarly, sophorolipids, a type of glycolipid biosurfactant embedded in plant-based composites, demonstrated antimicrobial efficiency against *C. acnes* (Cho et al., 2022).

Other compounds including indoles and farnesol, have been researched as potential antibacterial agents. Indoles are common natural compounds that have a bicyclic structure consisting of a pyrrole ring linked to a benzene ring. They can be found among humans as well as in bacteria and plants. Most remarkably, indoles serve as critical signaling molecules in bacterial and eukaryotic species alike. An investigation into 20 indoles’ antibacterial properties revealed that 3,3′-diindolylmethane (DIM) significantly inhibited planktonic cell growth and biofilm formation in *C. acnes*. DIM also suppressed multispecies biofilm formation and repressed biofilm-related genes in *C. acnes* suggesting a promising avenue for future research (Kim et al., 2022). Farnesol, with its anti-inflammatory and antimicrobial properties, showed promise in acne treatment through topical application, even though this compound was not directly tested on *C. acnes* biofilms but nonetheless, is worth mentioning as *C. acnes* biofilm has a significant role in acne formation (Wu et al., 2021).

Bacteriophages are viruses that infect and replicate within bacteria before lysing the bacterial cells. Since bacteriophages are highly specific to their host target which are bacteria, they can act as potential therapeutic agents against bacterial infections, including those caused by *C. acnes* (Fernández et al., 2019). One of the most crucial advantage of utilising phages is that phages are highly specific to their target bacterial species or even strains, which allow for precise targeting of pathogens like *C. acnes* while sparing other beneficial bacteria. Upon infecting a bacterial cell, phages inject their genetic material, which then hijacks the host bacterial machinery to replicate phage components. As a result, the bacterial cell lyses, releasing new phage particles to infect neighboring bacteria (Lin et al., 2022). An *in vitro* study using bacteriophages, PAP 1-1 and TCUCAP1, have shown promise in combating *C. acnes* infections. PAP 1-1 was shown to effectively control *C. acnes* proliferation, either as a single compound or in combination with other antimicrobial compounds such as nicin (Han, Khan & Moon, 2023). TCUCAP1, isolated from healthy volunteers’ skin, was shown to significantly reduce inflammatory lesions caused by multi-drug-resistant (MDR) *C. acnes* on mice model with induced skin inflammation (Lam et al., 2021). Efforts to formulate TCUCAP1 into a hydroxyethyl cellulose (HEC) cream suggest the potential of commercialising a product developed from bacteriophage as a novel antibacterial and antibiofilm agent to treat acne infections (Sá et al., 2023). A recent study on a novel lytic bacteriophage Corretto demonstrated a near-complete eradication of biofilms produced by multiple strains of *C. acnes* (Frantar et al., 2025). Since this isolate was obtained from human saliva, this *in vitro* study highlighted the potential of using this bacteriophage to treat persistent *C. acnes* infections in dental implants where conventional antibiotics fail.

The potential therapeutic benefits of the groups of natural agents based on effectiveness, safety and scalability has been summarized in Table 4. Overall, bacteriophages have the greatest therapeutic potential due to its superior effectiveness and specificity, despite moderate scalability challenges. Bacterial compounds and biosurfactants, on the other hand, offer a good balance of safety, effectiveness and scalability, which is more ideal commercially. Plant extracts provide the best scalability with proven safety, along with a moderate to high effectiveness. However, there is the issue of standardization and quality control as active compounds in plants vary among species, geographical distribution, climate, soil conditions, which would negatively impact commercial production. Nonetheless, there are many plant-based topical products available in the market, such as antibacterial creams and formulations which are neem-based, tea tree oil, tumeric-based products, all of which do not have available clinical data. In addition, liposome- and niosome-based formulations as antibiotics and anti-inflammatory agents for topical usage progressing into the late preclinical stages (Teo et al., 2025). Nonetheless, all the natural agents will require further testing and *in vivo* validation before clinical application can be considered.

**Table 4:** Comparison of natural agents as potential therapeutic agents. A summary table comparing the groups of natural substances based on effectiveness, safety and scalability as a potential therapeutic agent for the treatment of *C. acnes* infections.

**Natural agent category**	**Effectiveness**	**Safety**	**Scalability**	**Overall potential**
**Bacteriophages**	**Highest** - Near-complete biofilm eradication (Corretto); highly specific targeting; demonstrated efficacy in reducing MDR *C. acnes* inflammation	**High** - Highly specific, spares beneficial bacteria; no reported cytotoxicity	**Moderate** - Requires isolation and characterization; strain-specific; formulation challenges	**Excellent** - Most promising for targeted therapy
**Bacterial compounds**	**High** - DIM showed significant inhibition of biofilm formation and gene repression; biosurfactants achieved 82.5% biofilm reduction in MRSA	**High** - Natural metabolites; *Promicromonospora* extracts show anti-oxidant properties; generally non-toxic	**Moderate to High** - Fermentation-based production possible; requires optimization	**Very Good** - Strong candidate for development
**Plant extracts**	**Moderate to High** - *H. odoratissimum* shows strong anti-adherence; *C. americana* achieved 30–40% biofilm reduction; propolis downregulates attachment genes	**High** - Traditional use history; *Cotoneaster* species non-cytotoxic; propolis maintains skin microbiome	**High** - Abundant natural sources; established extraction methods	**Very Good** - Proven safety profile with moderate efficacy. Utilized in traditional medicine.
**Essential oils**	**Moderate to High** - Thyme and oregano EOs show potent bactericidal and antibiofilm effects; green tea modulates AI-2 production	**Moderate** - Skin sensitivity issues; potential medication interactions; volatility concerns	**Moderate** - Variable efficacy due to geographic/extraction factors; narrow spectrum activity	**Good** - Limited by variability and safety concerns
**Biosurfactants**	**High** - Glycolipid biosurfactant reduced biofilm by 82.5%; sophorolipids showed antimicrobial efficiency against *C. acnes*	**High** - Microbial-derived; biodegradable; generally biocompatible	**Moderate to High** - Microbial production scalable; requires optimization	**Very Good** - Promising but needs more specific *C. acnes* biofilm data
**Algae extracts**	**Moderate** - Shows antibacterial activity; ulvans modulate biofilm formation *in vitro*	**High** - Natural marine compounds; rich in bioactive polysaccharides	**High** - Abundant marine resources; sustainable harvesting	**Good** - Requires more investigation for biofilm-specific activity

## Dermatological Strategies

Dermatological strategy of using a 30% supramolecular salicylic acid peel, targets to redress the skin microbiota imbalance which is associated with acne. Clinical trials revealed a decreased abundance of *C. acnes* and Staphylococcus after treatment, suggesting potential therapeutic effects (Shao et al., 2023).

Another *in vitro* study evaluated a serum’s efficacy in eliminating the formation of biofilm by *C. acnes*. The active ingredients in this serum were Niacinamide (4%),10-Hydroxydecanoic acid, Sebacic acid, Ceramide 3, Phytosphingosine, Ceramide 6 II, Ceramide 1, Dimethylmethoxy Chromanol, D-d-tocopherol-containing compounds alongside emollients and occlusive agents to address skin barrier function. The serum was reported to effectively eradicate preformed biofilms, based on experimental data on *C. acnes* grown on both two-dimensional HaCaT keratinocyte monolayer cultures and three-dimensional tissue models including Reconstructed Human Epidermis (RHE) and Human Corneal Epithelium (HCE) (Sommatis et al., 2022). The results showed that the antibiofilm effect was concentration-dependent, indicating its potential to disrupt biofilm formation and mitigate acne pathogenesis (Sommatis et al., 2022).

An *in vitro* study on combating common scalp microorganisms like *C. acnes* using a non-cross-linked hyaluronic acid (HA) formulation, Hydro Deluxe, was evaluated. The HA formulation was assessed on a three-dimensional Reconstructed Human Epidermis (RHE) model infected with bacterial strains to induce an inflammatory response. The HA formulation was shown to exhibit potent antibiofilm activity without causing cytotoxicity and significantly reduced the expression of the pro-inflammatory marker Interleukin-8 (IL-8) (Sommatis et al., 2021).

Various nanoparticle techniques including antimicrobial peptides, photothermal treatment, and polymer-based drug delivery systems offer promising solutions against bacterial infections and biofilm-related resistance. Nanoparticles like liposomes and micelles increase drug bioavailability and enhance antibacterial properties against *C. acnes* biofilms. Liposomes loaded with DNase I and proteinase K have demonstrated antimicrobial and antibiofilm activities, while chitosan and hyaluronic acid nanoparticles could deliver clindamycin effectively to target sites, inhibiting bacterial growth (Himanshu et al., 2023; Singh et al., 2021).

Liposomes, known for their industrial-scale production and biocompatibility, are nanocarriers for nutraceuticals, and can serve as effective carriers for antibacterial agents. Enzyme-loaded cationic liposomes exhibit antibacterial and antibiofilm properties against *C. acnes*. Chitosan and hyaluronic acid nanoparticles loaded with clindamycin were shown to efficiently inhibit bacterial growth at the hair follicle unit, demonstrating potential for acne treatment (Makhlouf, Ali & Al-Sayah, 2023).

Nanomaterials combined with photothermal and photodynamic therapies, known as photo-nanodermatology, offer new avenues for acne treatment. Curcumin encapsulated in liposomal gold nanoparticles allow the delivery of plant extracts to the hair follicles. Once it reaches the target site, photothermal transduction will be induced, destroying sebaceous glands and exerting a bactericidal effect (Chakraborty, Narayanan & Gautam, 2022). However this was not tested directly on *C. acnes* biofilms and requires further research.

In the realm of acne treatment, nanoparticles offer innovative solutions targeting both the bacteria and inflammation associated with acne vulgaris. Immunomodulatory nanoparticles, exemplified by nitric oxide-releasing formulations, effectively combat *C. acnes* by inhibiting bacterial growth and suppressing the inflammatory response triggered by microbial invasion (Qin et al., 2015). Similarly, solid lipid nanoparticles used in conjunction with chitosan and tretinoin, demonstrate potent inhibition of *C. acnes* while ensuring compatibility with skin cells, showcasing their potential for topical application (Latter et al., 2019).

Recent studies explore the efficacy of plant-derived nanoparticles in acne management. Herbal topical gels incorporating silver nanoparticles derived from Neem (*Azadirachta indica*) and *Curcuma caesia* rhizome have been shown to exhibit significant antibacterial activity against *C. acnes*, presenting promising therapeutic options with minimal adverse effects (Baby et al., 2022; Chutoprapat, Kopongpanich & Chan, 2022).

Niosomes have the potential in acne treatment due to their ability to penetrate the skin effectively and release drugs continuously, In a clinical study, 45 Egyptian patients with acne vulgaris were treated using methylene blue niosomal hydrogel photodynamic therapy. It was reported the formulation effectively targeted acne lesions and reduce inflammation (El-Mahdy et al., 2020) with minimal adverse effects observed (Vyas, Kumar Sonker & Gidwani, 2014).

Antimicrobial peptides (AMPs) serve as key effectors in the innate immune response. A study by Kim et al. (2025) showed two peptides, DAP-7 and DAP-10, specifically designed against *C. acnes*, demonstrated significant anti-microbial activity against both antibiotic resistant and sensitive strains of *C. acnes*. These two novel AMPs disrupt the bacterial cell membranes of *C. acnes*, resulting in cell rupture. In addition, the AMPs were shown to reduce the release of pro-inflammatory cytokines in *C. acnes,* while exhibiting minimal toxicity to human cells. This research offers a potential new therapeutic approach for treating *C. acnes* infections, particularly important given the growing problem of antibiotic resistance (Kim et al., 2025). The dual action of these peptides—both antimicrobial and anti-inflammatory—are especially valuable for treating acne and prosthetic joint infections where *C. acnes* is implicated as the causative agent. In another study, Karnwal et al. (2023) has developed and synthesized a series of peptides P1: Rhamnolipids, P2: Sophorolipids, P3: Mannosylerythritol lipids (MELs), P4: Lipopeptides, P5: Trehalolipids, P6: Glycolipids. P1 to P6 with demonstrated antimicrobial efficacy against *C. acnes* and *Staphylococcus aureus*, suggesting their potential in combating dermal infections (Karnwal et al., 2023). All six peptides showed no cytotoxicity, antimicrobial, or antioxidative properties based on their structural characteristics. All the peptides with the exception of P1 and P3 showed different bioactivity when adsorbed onto bacterial cellulose scaffolds, demonstrating the potential to be develop into topical application (Golonka et al., 2021). In addition, C-type natriuretic peptide (CNP) potentially affect *C. acnes*, biofilm metabolism through zinc competition within the biofilms structure (Ovcharova et al., 2023).

Several cosmetic interventions targeting *C. acnes* biofilms have been developed including MPA-RegulTM (vegetal polysaccharide rich in gluconic acid), PS291^®^, UTW^®^, and ACNILYS^®^. These interventions have been found to suppress biofilm formation, decrease biofilm thickness and density, and disrupt biofilm structure, thus offering effective and promising solutions for managing *C. acnes*-related conditions (Fournière et al., 2020; Ionescu et al., 2015; Enault et al., 2014; Gannesen et al., 2019; Wunnoo, Saising & Voravuthikunchai, 2017). The properties of these four products are summarized in Table 5.

**Table 5:** Summary of the mechanisms of action, effects on biofilms of the four cosmetic interventions on *C. acnes* biofilms. Each of the four products indicates the mechanism of action and their effects on biofilms produced by *C. acnes*.

**Product**	**Mechanism of action**	**Effect on biofilm**	**Reference**
MPA-RegulTM	Inhibits bacterial adhesion to surfaces.	Suppresses biofilm formation, reduces thickness and density, decreases overall biomass associated with *C. acnes* infections.	Fournière et al. (2020)
PS291^®^	Disrupts biofilm structure, making it more susceptible to antimicrobial treatments.	Reduces biofilm formation and density, contributing to improved management of *C. acnes*-related conditions.	Gannesen et al. (2019)
UTW^®^ (UriageTM Thermal Water)	Disrupts the extracellular matrix of biofilms, leading to destabilization.	Reduces biofilm thickness and density, decreases biofilm formation.	Ionescu et al. (2015)
ACNILYS	Inhibits key processes involved in biofilm development.	Suppresses biofilm formation, decreases thickness and density.	Wunnoo, Saising & Voravuthikunchai (2017)

## Innovative Antibiofilm Devices

Recent advancements in electrochemical devices have shown promising potential in combating *C. acnes* biofilms and other microbial biofilms without relying on antibiotics.

One such innovation is the electrochemical bandage (e-bandage) composed of carbon fabric, that continuously releases low levels of hydrogen peroxide (H_2_O_2_) to target bacterial biofilms. The e-bandage was tested on *C. acnes* as individual and co-culture with results showing a reduction in viable cell counts *in vitro* study, after 48 h of treatment. This demonstrates the potential of the e-bandage as an antibiotic-free treatment for chronic wound infections (Raval et al., 2021).

**Figure 3:**
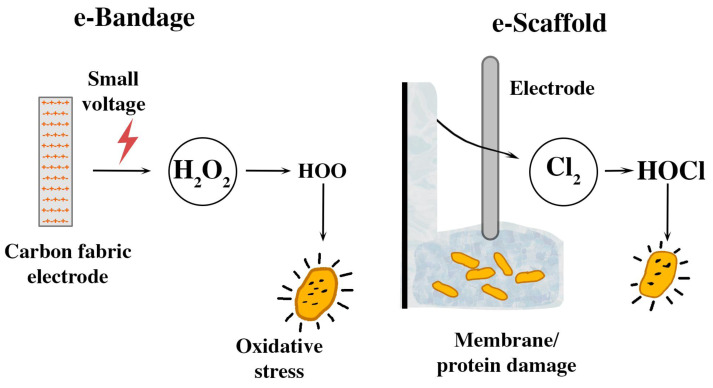
Schematic representations of the e-bandage and electrochemical scaffolding.

A new electrochemical scaffold, called an e-scaffold, can target microbial biofilms by continuously releasing safe dosages of HOCl. This device was tested against biofilms developed from 33 single bacterial isolates, and combinations of two bacterial species, including those resistant to antibiotics. When energized at 1.5 V, the e-scaffold effectively reduced the number of viable bacterial cells in both single-species and dual-species biofilms, highlighting its efficacy against antibiotic-resistant bacteria. The HOCl production was achieved by electrochemically converting chloride ions to chlorine, which then formed HOCl in water. These findings suggest that electrochemical devices utilizing H_2_O_2_ and HOCl have promising applications in combating microbial biofilms and may offer alternative strategies for managing infections (Flurin et al., 2021). Figure 3 illustrates the mechanism of action of these innovative electrochemical devices against biofilms. The e-bandage and the e-scaffold offer an antibiotic-free strategy for targeting *C. acnes* and other microbial biofilms. However, these innovative devices require further refinement to optimize its safety, efficacy, and clinical usability before it can be integrated as therapeutic options in the clinical setting. After establishing this foundation, pilot studies targeting wound care and implant-related infections, especially chronic cases, can be initiated.

Polystyrene surfaces coated with plasma, serum, or albumin, were studied for their impact on *C. acnes* adhesion and biofilm formation. Protein pre-adsorption or simultaneous addition with bacterial cells significantly reduced *C. acnes* attachment and biofilm formation compared to controls. Blood components with calcium was found to be able to inhibit the bacterial adhesion process whilst zinc was reported to reduce biofilm formation of *C. acnes*. Interestingly, *C. acnes* cells did not adhere to erythrocytes, suggesting a preference for environments with lower plasma content (Polyudova, Eroshenko & Korobov, 2018). The real-world impact of surface coatings and blood component treatments in actual implant settings need to be further investigated to bridge the gap between laboratory findings and clinical applications.

## Conclusions

*C. acnes* produces biofilms that contribute to its virulence and ability to establish chronic infections, particularly on indwelling medical devices. Understanding how these biofilms develop is crucial for finding better ways to prevent and treat these infections. Researchers are exploring new treatment options, particularly because antibiotic resistance is making current treatments less effective.

Although some studies have shown promising results with plant-based and nanomaterial treatments, there are still challenges. Many studies use lab models that do not fully reflect real-life conditions, and lack further validation in the way of pre-clinical and clinical trials to accurately measure treatment effectiveness. Additionally, plant-based treatments face issues such as variability in their active ingredients, difficulty in creating stable formulations, and slow regulatory approval. Nanomaterials also raise concerns on its safety, environmental impact, and the risk of bacteria developing resistance over time (Ngoepe, Schoeman & Roux, 2025).

To improve future treatments options, *in vitro* research need to be developed and further refined into *in vivo* studies using animal models and eventually in actual patients. Subsequently, larger-scale clinical trials with heterogeneous participant groups are required to establish the reliability and clinical effectiveness of these therapeutic approaches against *C. acnes*, with particular focus on deep-tissue infections and implant-associated biofilm formation. Standardizing plant extracts, optimizing dosages, and understanding how these treatments work will help refine them. An option to minimize the variability of plant sources from different soil types and geographic regions would be the use of large-scale tissue culture that can control these parameters and provide plant material with standardized chemical composition, reliable potency, and batch-to-batch consistency. Combining different therapies may also enhance their effectiveness. Overcoming these challenges can make these alternative treatments to become valuable options for managing *C. acnes* infections which would prove beneficial for clinicians, including dermatologists.

In conclusion, despite the fact that these studies offer promising insights into acne biofilm-related infection treatments, further research is needed to bridge the gap between laboratory findings and clinical applications. With the consideration of many dynamic factors, larger clinical trials with diverse participants is crucial to confirm the effectiveness and safety of each of these approaches. Standardized extracts, optimized dosing, and thorough studies on how these extracts work will guide future drug development. In addition, overcoming formulation challenges and exploring combination therapies with other acne treatments are promising paths to overcome the incidence of drug-resistance when treating *C. acnes* infections. By addressing these points, such therapies and techniques will have the potential to become a strong addition to the *C. acnes* infection treatment regimen, offering patients wider choices for managing their condition.

##  Supplemental Information

10.7717/peerj.20652/supp-1 Supplemental Information 1 PRISMA checklist

10.7717/peerj.20652/supp-2 Supplemental Information 2 Flow diagram depicting identification of studies via databases

## Additional Information and Declarations

### Data Availability

The following information was supplied regarding data availability: This is a literature review.
