# Supplementary material for: Battling biofilms: evaluating selected agents against Cutibacterium acnes—a review
Source: PeerJ. 2026 Jan 28;14:e20652. doi: 10.7717/peerj.20652 (PMC12860307; doi:10.7717/peerj.20652)
Supplement: Supplemental Information 2 [file peerj-14-20652-s002.docx]

**Identification of studies via databases and registers**

Records removed *before screening*:

Duplicate records removed (n =14 )

Records marked as ineligible by automation tools (n =20)

Records removed for other reasons (n =0)

Records identified from*:

Databases (n =139)

Registers (n = 0)

**Identification**

Records screened

(n =125)

Records excluded**

(n =20)

Reports sought for retrieval

(n =105)

Reports not retrieved

(n = 0)

**Screening**

Reports excluded:

Did not assess biofilm activity (n = 12)

Used synthetic or non-plant compounds (n =10)

Data report was not sufficient (n=5)

Reports assessed for eligibility

(n =78)

Studies included in review

(n = 131)

Reports of included studies

(n =157)

**Included**

*Consider, if feasible to do so, reporting the number of records identified from each database or register searched (rather than the total number across all databases/registers).

**If automation tools were used, indicate how many records were excluded by a human and how many were excluded by automation tools.

Source: Page MJ, et al. BMJ 2021;372:n71. doi: 10.1136/bmj.n71.

This work is licensed under CC BY 4.0. To view a copy of this license, visit <https://creativecommons.org/licenses/by/4.0/>
